# Wound in the Stomach Treated by Opium

**Published:** 1860-05

**Authors:** James Cody

**Affiliations:** Watertown, Wis.


ARTICLE 3.

### WOUND OF THE STOMACH TREATED BY LARGE DOSES OF OPIUM.

BY DR. JAMES CODY, OF WATERTOWN, WIS.

I was called in haste on the night of November 27th, last, at 11½ o'clock, P. M., to see Wm. Forsyth, aged nineteen years, who, a few minutes before, was reported to have received a stab in the left side, in an affray at a saloon. I found him lying on the floor; face pale and exsanguinous; extremities cold; pulse very small; he complained of feeling very weak and faint. On examination I found an incised wound on the left side, between the ninth and tenth ribs, seven and a half inches from the sternum and nine inches from the spine. The wound was about one inch in length, and parallel with the ribs; through the wound a small portion of the stomach had protruded. This was returned, not into the cavity of the abdomen, but left resting in the muscular tissue between the ribs; the edges of the wound was now brought together, one suture taken, and strips of adhesive plaster snugly applied, a bandage closely drawn around the chest, completed the dressing.

One-third of a grain of morphine was now given, with directions to repeat the same every two hours until he became fully under its influence.

28th, 8 A. M.—He has taken three doses of morphine, and is fully under its influence, breathing very heavily, and is aroused with difficulty; when aroused complains of no pain, but is very thirsty; stomach considerably distended and resonant on percussion; on moving him in bed he vomited freely; pulse 140 per minute; is to continue morphine, and drink ice water.

8 P. M.—Stomach greatly distended; but little or no fulness of abdomen, (except over the region of the stomach;) high fever; pulse 140, hard and bounding; breathing difficult; when roused up is delirious. Bled him copiously, which reduced the pulse; is to continue the morphine during the night; also the ice water as drink; apply ice water to head.

29th, 8 A. M.—Morphine was omitted twice during the night, for the reason that he became narcotised (it will be remembered that he had taken one-third of a grain every two hours,) stomach greatly distended; some tympanites of the abdomen; pulse 140, but softer than yesterday; is delirious when roused up; complains of no pain; says he dreams very much, but when asked what he dreams says he forgets; is to continue morphine, also ice water for drink; is to have an injection to unload the bowels.

8 P. M.—Has had two dejections; stomach about the same; no fulness of abdomen; complains, for the first time, of pain in the region of the stomach; on pressure over the stomach he flinches. Applied a blister over the stomach; continue morphine; is to drink ice water.

30th, 8 A. M.—Morphine was omitted at 11 and 4 o'clock. The pulse is 130; not so delirious when roused up, this morning; says he feels a burning sensation in the stomach; no appetite; is thirsty and drinks a large quantity of water The stomach keeps greatly distended yet, is to continue morphine as heretofore.

8 P. M.—Was rather more restless to-day; complained of pain in the head; attempted to get up several times; some twitching of the tendons of the fore arms; says he feels faint; is not so thirsty; continue morphine; also give injection of beef tea.

December 1st, 8 A. M.—Distention of the stomach the same; complains of slight soreness and burning in it; pulse

130 and small; some pain in the head; is not very thirsty; says he dreams much—dreams are pleasant; some subsultus tendinum; took morphine $\frac{1}{4}$ gr. every three hours during the night; is to continue the same to-day; also injections of beef tea, one-half pint every four hours.

8 P. M—Pulse 130 small; appears very drowsy, but is perfectly rational when aroused; complains of no pain; continue treatment the same.

2d, 8 A. M.—Pulse 130; reported to have rested well during the night; his condition is about the same as yesterday; continue treatment the same.

8 P. M.—The same as in the morning. Gave the morphine only every four hours; injections the same.

3d, 8 A. M.—Rested well last night; is evidently improving; pulse 120 and small; mind clearer; stomach less distended. Is to take arrow root to-day, also continue injections of beef tea, and give morph. $\frac{1}{4}$ gr. every four hours.

8 P. M.—Complains of feeling very weak; arrow root was well relished, and rested on the stomach well; pulse 120, and of better character; the fulness of the stomach is evidently decreasing; continue arrow root for drink, also morph.; brandy to be added to the injections.

4th, 8 A. M.—Stomach less distended; spent a good night; seems improving, continue treatment.

8 P. M.—Appears much stronger; expresses himself as doing well.

5th, 8 A. M.—Continues to improve.

6th, 8 A. M.—The same as yesterday.

7th, 8 A. M.—Is improving rapidly; pulse 100; takes less morphine—$\frac{1}{4}$ gr. every six hours.

8th, 8 A. M.—Continues improving; has an appetite, and wants to eat; is to have rice.

9th.—Reports himself as well this morning; needs no medicine.

10th, 8 A. M.—Discharged as cured.

